# State Anxiety Subjective Imbalance and Handicap in Vestibular Schwannoma

**DOI:** 10.3389/fneur.2016.00101

**Published:** 2016-07-13

**Authors:** Yougan Saman, Lucie Mclellan, Laurence Mckenna, Mayank B. Dutia, Rupert Obholzer, Gerald Libby, Michael Gleeson, Doris-Eva Bamiou

**Affiliations:** ^1^Institute of Neurology, National Hospital for Neurology and Neurosurgery, UCL, London, UK; ^2^Nelson R. Mandela School of Medicine, UKZN, Durban, South Africa; ^3^Neuro-otology Department, National Hospital for Neurology and Neurosurgery, London, England; ^4^Royal National Throat, Nose and Ear Hospital, London, UK; ^5^Centre for Integrative Physiology, University of Edinburgh, Edinburgh, UK; ^6^ENT and Skull Base Department, Guys Hospital, London, UK; ^7^ENT and Skull Base Department, Kings College Hospital, London, UK; ^8^Neurogastroenterology Group, Queen Mary University of London, London, UK; ^9^Neuro-otology and Skull Base Department, National Hospital for Neurology and Neurosurgery, London, UK; ^10^Ear Institute, UCL, London, UK

**Keywords:** balance, handicap, anxiety, vestibular schwannoma, cognitive behavioral therapy

## Abstract

**Introduction:**

Evidence is emerging for a significant clinical and neuroanatomical relationship between balance and anxiety. Research has suggested a potentially priming effect with anxiety symptoms predicting a worsening of balance function in patients with underlying balance dysfunction. We propose to show that a vestibular stimulus is responsible for an increase in state anxiety, and there is a relationship between increased state anxiety and worsening balance function.

**Aims:**

(1) To quantify state anxiety following a vestibular stimulus in patients with a chronic vestibular deficit. (2) To determine if state anxiety during a vestibular stimulus would correlate with the severity of chronic balance symptoms and handicap.

**Methods:**

Two separate cohorts of vestibular schwannoma (VS) patients underwent vestibular tests (electronystagmography, cervical and ocular vestibular evoked myogenic potentials, and caloric responses) and questionnaire assessments [vertigo handicap questionnaire (VHQ), vertigo symptom scale (VSS), and state-trait anxiety inventory (STAIY)]. Fifteen post-resection VS patients, with complete unilateral vestibular deafferentation, were assessed at a minimum of 6 months after surgery in Experiment 1 (Aim 1). Forty-five patients with VS *in situ* formed the cohort for Experiment 2 (Aim 2). Experiment 1: VS subjects (*N* = 15) with a complete post-resection unilateral vestibular deafferentation completed a state anxiety questionnaire before caloric assessment and again afterward with the point of maximal vertigo as the reference (Aim 1). Experiment 2: state anxiety measured at the point of maximal vertigo following a caloric assessment was compared between two groups of patients with VS in situ presenting with balance symptoms (Group 1, *N* = 26) and without balance symptoms (Group 2, *N* = 11) (Aim 2). The presence of balance symptoms was defined as having a positive score on the VSS–VER.

**Results:**

In Experiment 1, a significant difference (*p* < 0.01) was found when comparing STAIY at baseline and at the peak of the subjective vertiginous response in post-resection patients with a unilateral vestibular deafferentation. In Experiment 2, VS *in situ* patients with balance symptoms had significantly worse state anxiety at the peak vertiginous response than patients without balance symptoms (*p* < 0.001), as did patients with a balance-related handicap (*p* < 0.001).

**Conclusion:**

Anxiety symptoms during a vestibular stimulus may contribute to a priming effect that could explain worsening balance function.

## Introduction

Dizziness, a frequent symptom at a population level, has a reciprocal relationship with anxiety: dizziness can be a symptom of a generalized anxiety disorder, whereas, conversely, secondary anxiety can be a frequent feature of a recurrent or chronic vestibular disorder (1, 2). The 2008 National Health Service Interview of American households, completed by 20,950 out of 21,780 participants ≥18 years, found an annual prevalence of vestibular vertigo of 8.4%, with the presence of vestibular vertigo trebling the odds of anxiety, panic disorder, and depression (3). The core neuroanatomical site for this interaction is the parabrachial nucleus, i.e., the substrate for panic and anxiety disorders (4). This nucleus is at the center of a network linking the vestibular nuclei with the amygdaloid nucleus, the infralimbic cortex, and the hypothalamus and subserving visceral, somatic, and vestibular information processing, which may mediate avoidance, conditioned fear, and anxiety responses to vestibular sensory information (5).

Clinically, the mechanisms for the complex dizziness and anxiety interaction remain unclear (1, 6, 7). A longitudinal study of 101 patients assessed after a 7-month interval found that autonomic symptoms and somatic anxiety were the best predictors of increased severity of vertiginous symptoms over time, while somatic anxiety symptoms (i.e., muscle tension, back pain, poor concentration and memory, and chest pain) were predictive of handicap (8, 9). It was postulated that autonomic symptoms initially associated with vestibular dysfunction during a vertiginous attack cause anxiety and further physiological arousal, which worsens the vertigo in a vicious circle (9). Another study reported that 1 year after an episode of vestibular neuritis (VN), the extent of a subject’s anxiety-related cognitions and body sensations predicted their vertigo severity independently of the degree of vestibular deficit on posturography. It was argued that the changed, threatening interpretation of the bodily sensation of dizziness that occurs in certain vulnerable individuals after a vestibular illness could be responsible for the chronicity of the dizziness, even in the absence of an “active” vestibular deficit (10). In addition, the presence and extent of anxiety-related cognitions and body sensations within the first week to sixth month period after VN incrementally predict the development of a panic disorder at 2 years (11). The patient’s psychological structure, however (assessed 4–8 weeks after the VN), did not predict the development of panic disorder (12).

In vestibular patients, anxiety symptoms may thus be part of a somatopsychic process as imbalance could lead to fear, panic, and avoidance. Psychosomatic anxiety symptoms, however, could also lead to a worsening of balance function (13). There is thus a growing appreciation in the literature about the need to manage anxiety more “aggressively” from the onset of the vestibular disorder, e.g., by cognitive behavioral therapy (CBT) (14) or other psychiatric treatment (15) together with physical therapy. It is argued that such early instigated psychological/psychiatric management may prevent what is potentially a priming effect resulting in increasing anxiety with subsequent vestibular episodes and consequently an increase in balance symptom severity and handicap. Psychologists and psychiatrists are thus increasingly forming part of the multidisciplinary team treating patients with balance disorders. Further research is needed to clarify this relationship and to identify other factors such as whether stress levels related to anxiety may also play a mechanistic role in ongoing balance dysfunction (16).

State anxiety can be described as fearful arousal at a given point in time and includes autonomic, behavioral, and cognitive components, whereas trait anxiety is one’s inherent reactivity to perceived or anticipated threat (17, 18). State anxiety has been shown to affect posture, gait, and gaze. At heights, normal subjects can experience increased state anxiety, autonomic activation, and changes in cognitive appraisals about their postural control with postural and gait alterations. In addition, the effects of state anxiety on postural control may also be related to trait anxiety. However, the interactions between state anxiety and postural control in patients with vestibular deficits remains poorly reported (18).

Similarly, both threat and trait anxiety affect gaze fixation in normal subjects. Both state and trait anxiety create a gaze bias toward threatening stimuli in the visual field with higher state anxiety contributing to quicker fixation while higher trait anxiety was associated with slower disengagement. Increased state anxiety also produced a “Stroop effect” on gaze control. Following acute vestibular insults, persistent changes have been noted in the subjective visual vertical during elevated state anxiety, but not trait anxiety (17).

In this study, we set out to investigate the interaction between state anxiety experienced during acute vestibular stimulation, chronic balance symptoms, and handicap reported by the patients in the vestibular schwannoma (VS) cohort.

The first experiment attempts to establish the effect of vestibular stimulation (using a caloric test) on a well-validated measure of anxiety levels at a specific point in time, the state anxiety questionnaire (STAI-Y1), in a cohort of post-resection VS patients with a complete unilateral vestibular deficit on vestibular tests.

The second experiment assesses the relationship between state anxiety levels during vestibular stimulation and the severity of chronic balance symptoms (VSS–VER) and patient reported handicap using the vertigo handicap questionnaire (VHQ) in a cohort of VS in situ patients. We hypothesized that:

1. Experiment 1: there would be a significant increase in state anxiety following a vestibular stimulus in patients with a chronic unilateral vestibular deficit, and
2. Experiment 2: state anxiety during a vestibular stimulus would correlate with the severity of chronic balance symptoms and handicap.

## Materials and Methods

The study was approved by the Central London Research Ethics Committee-3 (REC reference 10/H0716/58). All participants were provided with a Patient Information Sheet prior to giving written informed consent.

All subjects completed a routine neuro-otological and questionnaire assessment.

### Electronystagmography

Horizontal direct current electronystagmography (ENG) was recorded using an EasyGraf recorder. This included recording of gaze (center and ±30°) with/without optic fixation, saccades at 0° and ±30°, smooth pursuit at 0.2, 0.3, and 0.4 Hz across 30° right and left of the mid position, optokinetic responses to a full-field striped curtain rotated at 40°/s, and sinusoidal rotation at 0.2 Hz with/without fixation.

### Caloric Testing

Bithermal caloric testing was undertaken using videonystagmography (each ear irrigated with water for 30 s at 30 and 44°C). Results were based on the degree of canal paresis (CP) determined by Jongkees formula. A CP >20% was considered abnormal.

### Cervical Vestibular Evoked Myogenic Potentials and Ocular Vestibular Evoked Myogenic Potentials Testing

Vestibular evoked myogenic potentials (VEMPs) were recorded and analyzed using a synergy evoked potential system. The cervical vestibular evoked myogenic potential (cVEMP) stimulus was delivered *via* TDH 49 headphones and consisted of 500 Hz alternating polarity tone bursts with a duration of 6 ms (2 ms rise/fall) delivered at a rate of 4.7/s for 200 stimulus presentations. Bandpass filtering was set to 20–2000 Hz, and a Blackman filter was used. A custom built voltmeter was used for biofeedback. Two consecutive runs were recorded for a stimulus of 120 dB SPL for each ear and if no clear response was obtained then this was increased to 125 dB SPL and repeated. The parameter of interest was the inter-aural asymmetry ratio (departmental normative data >38% is significant).

The ocular vestibular evoked myogenic potentials (oVEMPs) bone-conducted vibration (BCV) stimuli was delivered by a hand-held bone-vibrator (Bruel and Kjaer 4810 minishaker and 2718 power amplifier) at Fz. The stimulus delivered was a 500-Hz tone burst of alternating polarity delivered at a repetition rate of 11/s, at 115 dB SPL, with duration of 6 ms (2–2–2 rise–plateau–fall) and with a low filter of 20 Hz and high filter of 500 Hz. The peaks n10 and P15 were marked, and the peak-to-peak amplitude between the first negativity and positivity was used as the parameter of interest to calculate inter-ocular asymmetry (departmental normative data >34% is significant).

### Vertigo Handicap Questionnaire

The VHQ is a validated tool assessing the physical, emotional, and practical impact of balance-related symptoms with 25 items. Disability or handicap are rated on a numeric scale from 0 (never or no handicap) to 4 (always or maximum handicap). To calculate the total handicap score, responses to items 1–25 of the VHQ are added after first reversing the scores on asterisked items resulting in a total score out of 100 (19).

### Vertigo Symptom Scale

This questionnaire contains 36 items and produces 2 subscales reflecting the severity of balance symptoms (VSS–VER) and anxiety symptoms (VSS–SA) as a function of frequency and when appropriate, duration (20). VSS responses are considered over a year or from the onset of vertigo and scaled from 0 = never, 1 = a few times (1–3 times/year), 2 = several times (4–12/year), 3 = quite often (on average more than once a month), and 4 (on average more than once a week).

### The State-Trait Anxiety Inventory

The state-trait anxiety inventory (STAI-Y1) measures general anxiety at a point in time using a 20-item scale based on a 4-point Likert scale. An attainable score ranges from 20–80 with the higher score suggesting greater anxiety. It is a well-validated scale that takes 10 min to complete (21).

Vestibular schwannoma patients were recruited from Guy’s Hospital and the National Hospital for Neurology and Neurosurgery.

Patients were included in the study if:

Experiment 1 cohort: participants had undergone VS resection at least 6 months prior to the study and had a complete unilateral vestibular deficit on the operated side as judged on the basis of absent caloric response to either warm or cold water and absent cVEMP and oVEMP tracings.
Experiment 2 cohort: participants had been diagnosed previously with a unilateral VS, were under observation using repeated MRI and showed abnormal vestibular function on Bithermal Calorics, cVEMP, or oVEMP.

All patients completed the VSS–VER and the VHQ, as well as the STAI-Y1, at the point of maximal vertigo during caloric stimulation as determined by the patient.

#### Experiment 1. State Anxiety before and during a Vestibular Stimulus in Post-Resection VS Patients

Fifteen postoperative VS patients were recruited. Tumor size ranged from 4 to 30 mm with a mean of 18 mm (SD 9) measured as the widest diameter in the cerebellopontine angle in the axial plane. Patients presented with a unilateral complete deafferentation (on caloric, cVEMP, and oVEMP) and were, therefore, homogeneous with regard to their vestibular deficit.

Subjects completed the STAI-Y1 before the caloric testing as a baseline. Both ears were irrigated with both warm and cold water as in a standard Bithermal Caloric protocol. Immediately after the test was complete, patients were sat up and asked to complete the STAI-YI again with the point of maximal vertigo experienced subjectively during the caloric, as the reference point irrespective of whether it was during the hot or cold water irrigation.

#### Experiment 2. State Anxiety during Caloric Stimulation and Correlation with Balance Symptom Severity (VSS–VER) and Handicap (VHQ) in VS Patients Observed with MRI

Forty-five patients were assessed (21 F, 24 M) with a mean age of 50.6 (SD 10.6). Tumor size ranged from intracanalicular to 25 mm measured as the widest diameter in the cerebellopontine angle in the axial plane. Eight out of 45 patients had no identifiable abnormality on vestibular testing. Twenty-three patients had abnormal caloric function with 6 having complete loss of caloric function while 24 patients had an abnormal cVEMP. Out of 43 patients who completed oVEMP testing, 15 had an abnormality. Three patients had a complete loss of caloric function with abnormal cVEMPs and oVEMPs. Both ears were irrigated with both warm and cold water as in a standard Bithermal Caloric protocol. Immediately after the test was completed, patients were sat up and asked to complete the STAI-YI with the point of maximal vertigo experienced subjectively during the caloric as the reference point irrespective of whether it was during the hot or cold water irrigation.

Patients with a VS *in situ* were divided into groups based on the VSS–VER, i.e., Group 1 presented with no balance symptoms (VSS–VER = 0), and Group 2 presented with some degree of balance dysfunction such as dizziness, unsteadiness or vertigo, or a combination of symptoms (VSS–VER > 0). STAI-Y1 scores were compared between the two groups and correlated with VSS-VER and VHQ.

## Results

### Experiment 1: State Anxiety before and during a Vestibular Stimulus in Post-Resection VS Patients

Patient characteristics are summarized in Table 1. Time post surgery ranged from 6 to 61 months with a mean of 18 months (SD 15). All subjects presented with a complete unilateral loss of caloric function, and no identifiable trace on either cVEMPs or oVEMPs. One patient showed saccadic intrusion on smooth pursuit testing during the ENG central test battery. Two patients scored 0 on the VSS–VER. There was a significant correlation between the post-caloric STAI-Y1 and the VHQ (*p* < 0.05), but not the VSS–VER.

**Table 1:** **Experiment 1 – state anxiety before and at the point of maximal vertiginous response during caloric stimulation**.

		Count	Mean	SD	Median
Sex	Female	8			
	Male	7			
Age			50.33	9.24	49.00
VSS–VER			10.93	12.5	8
VHQ			18	17.3	15
State anxiety pre-caloric			32.33	13.26	25.00
State anxiety at peak caloric response			47.40	17.27	48.00

**Figure 1:**
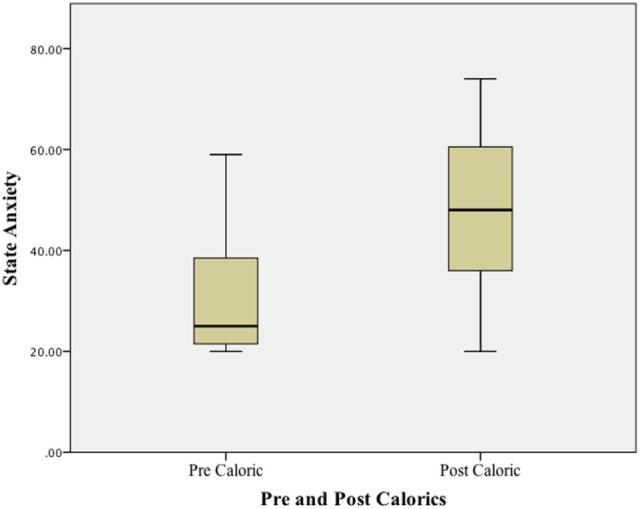
**State anxiety before and after caloric stimulation in post-resection VS patients**.

Using a Wilcoxon Signed ranks test, a significant difference (*p* < 0.01) was found when comparing state anxiety (STAI-Y1) at baseline (before any vestibular stimulus) and at the peak of the subjective vertiginous response during the caloric stimulus. STAI–YI values are tabulated in Table 1 and illustrated in boxplots in Figure 1. The change in state anxiety (STAI-Y1 at peak response − STAI-Y1 pre-testing) correlated significantly with the STAI-Y1 peak response (*r* = 0.69, *p* < 0.01).

### Experiment 2: State Anxiety (STAI-Y1) during Caloric Stimulation, Balance Symptom Severity (VSS–VER), and Handicap in VS Patients Being Observed with MRI

Questionnaire assessments relating to balance dysfunction or anxiety were not significantly different in patients with normal (8 patients) vs. abnormal (37 patients) vestibular test results.

Further analysis was confined to the 37 patients presenting with an abnormality on vestibular testing. Eleven out of these 37 patients scored 0 on the VSS–VER and, therefore, had no balance symptoms, with the remaining 26 reporting a range of balance symptoms of varying severity.

**Figure 2:**
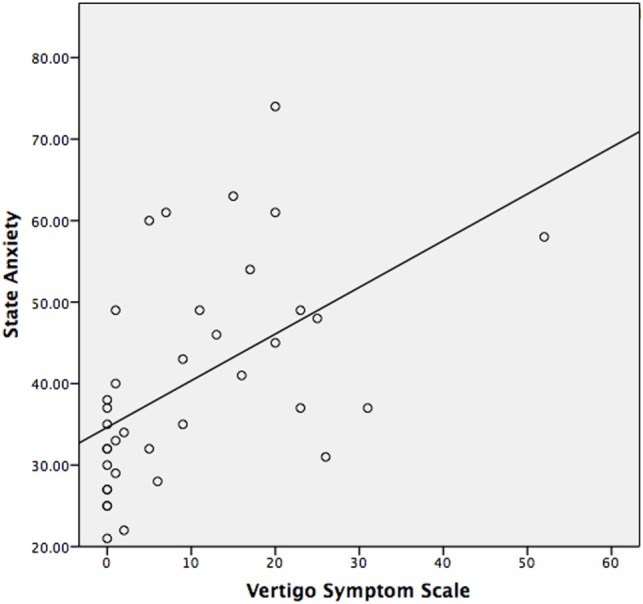
**State anxiety correlated with the VSS–VER in VS patients observed with MRI**.

**Figure 3:**
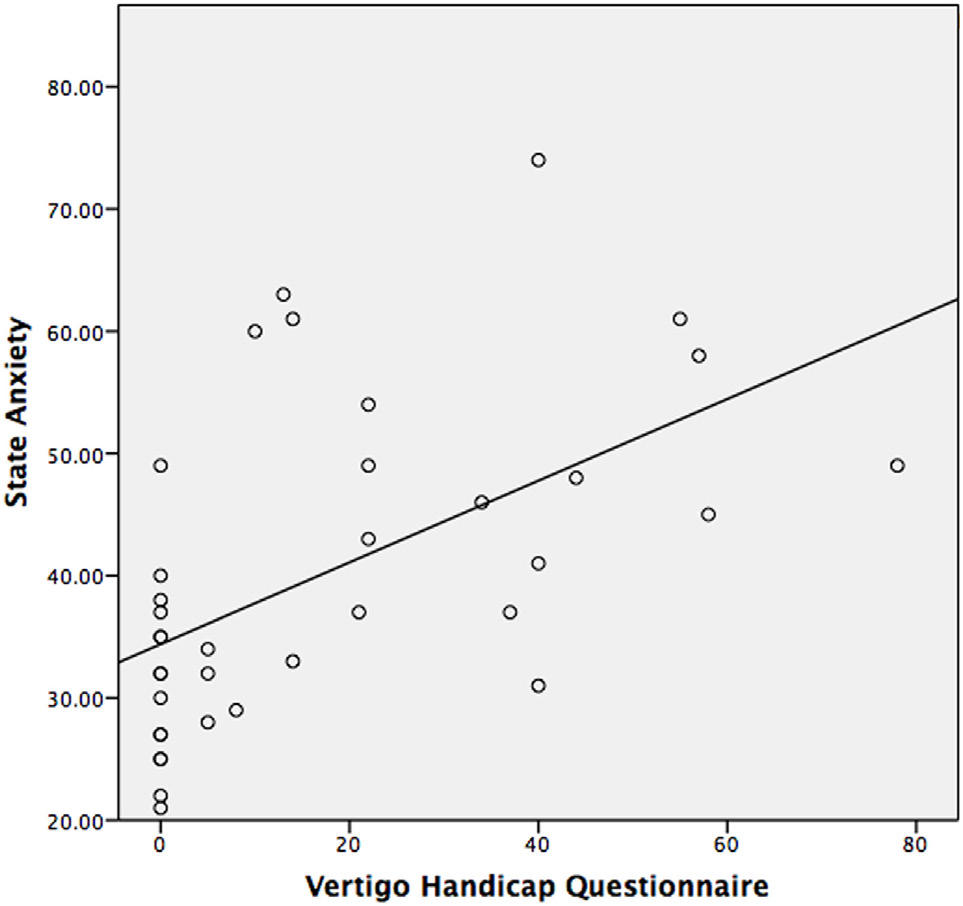
**State anxiety vs. VHQ in VS patients observed with MRI**.

The median STAI-Y1 score for Group 1 (VSS–VER = 0) was 30 and Group 2 (VSS–VER > 0) was 44 (Table 2). A strong correlation (Spearman’s rho) was found between peak caloric stimulation STAI-Y1 scores and the VSS–VER (*r* = 0.61, *p* < 0.001) (Figure 2) and the VHQ (*r* = 0.63, *p* < 0.001) (Figure 3).

**Table 2:** **Experiment 2 – state anxiety in patients with and without balance symptoms**.

Balance symptoms							
No				Yes			
State anxiety peak vertiginous response				State anxiety peak vertiginous response			
Mean	SD	Median	Count	Mean	SD	Median	Count
29.9	5.43	30.00	11	44.6	12.9	44.00	26

**Figure 4:**
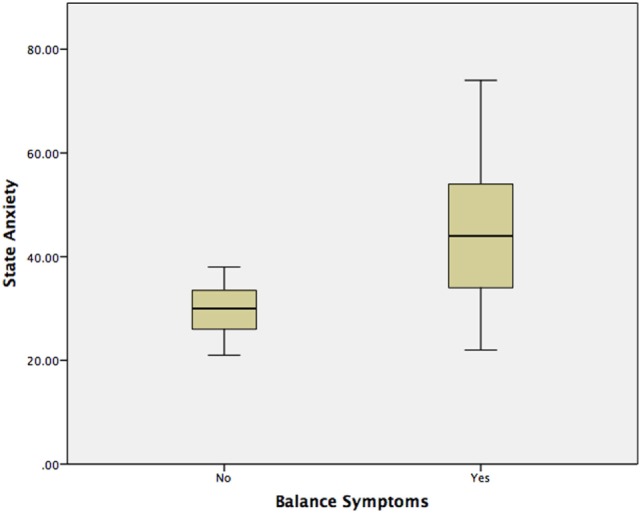
**State anxiety vs. balance symptoms in VS patients observed with MRI**.

**Figure 5:**
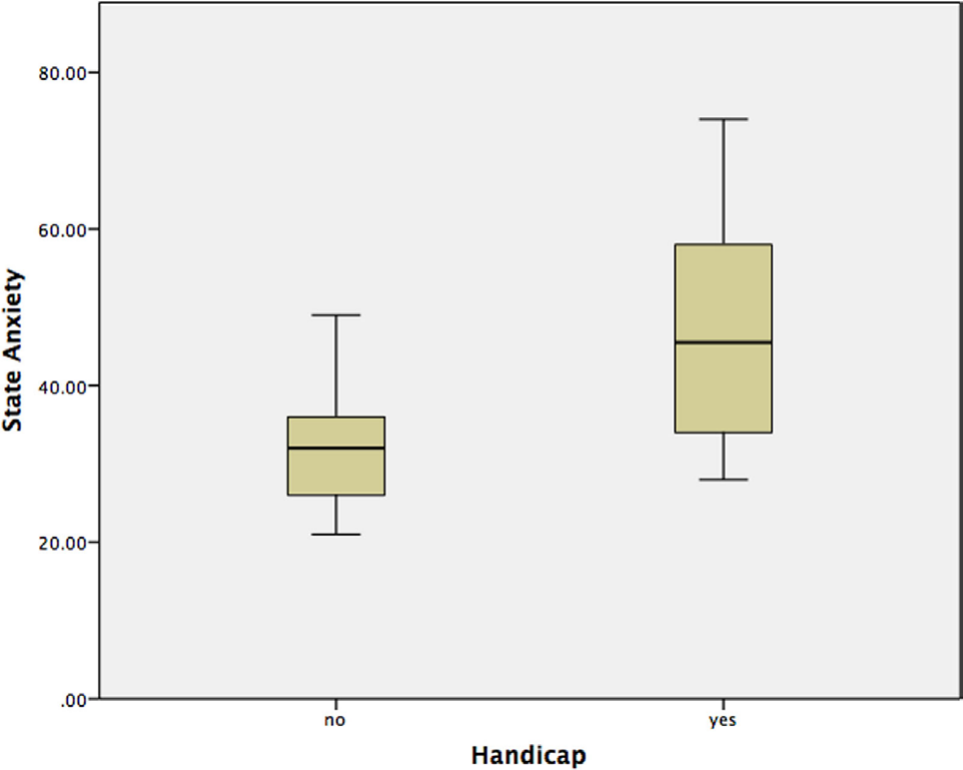
**State anxiety vs. handicap (boxplot) in VS patients observed with MRI**.

Using a Mann–Whitney test, Group 2 had significantly worse state anxiety at the peak vertiginous response when compared with Group 1 (*p* < 0.001) (Figure 4), as did patients with a balance-related handicap (score >0 on the VHQ) (*p* < 0.001) (Table 3; Figure 5).

**Table 3:** **State anxiety in patients with and without balance-related handicap**.

Balance-related handicap							
No				Yes			
State anxiety peak vertiginous response				State anxiety peak vertiginous response			
Mean	SD	Median	Count	Mean	SD	Median	Count
31.7	7.6	32.00	15	46	12.9	46.00	22

## Discussion

This study has attempted to further delineate the relationship between anxiety, balance symptoms, and handicap in the VS population. We set out to determine if a vestibular stimulus resulted in a change in state anxiety in VS patients with a chronic vestibular deficit and if this was related to the severity of symptoms or handicap. VS is a slow growing tumor that gradually produces some degree of vestibular deafferentation, with the majority of patients apparently well compensated at presentation. Several patients, however, still complain of balance symptoms with some being quite severely disabled, and the literature does suggest a role for anxiety as a significant factor (22). In this and our previous studies, our aim has been to better understand the role of anxiety in patients with balance dysfunction in order to choose appropriate rehabilitation strategies. Increasingly, the majority of patients in our setting are offered a “watchful waiting” approach, a strategy involving observation with serial MRI as most tumors do not demonstrate the potential for significant growth. Therefore, understanding the interaction between anxiety and imbalance in this group of patients with varying degrees of vestibular loss, which in some cases may be progressive, is of significant clinical importance.

In patients with differing pathologies, Eckhardt-Henn et al. found that the relationship between balance symptoms and anxiety was far more significant in patients with Menière’s disease and vestibular migraine as opposed to patients with benign positional paroxysmal vertigo (BPPV) and VN and argued that it was the unpredictability and uncontrollability of symptoms that strongly influenced the presence of anxiety in these patients (23). Despite the absence of unpredictable vertigo attacks, we have shown previously that anxiety symptoms are significantly greater in VS patients with balance symptoms when compared with healthy controls while anxiety symptoms, ambulant postural stability, and the severity of balance symptoms are all significant predictors of handicap (24). In this study, we argued that even in the absence of recurrent persistent unpredictable vertigo attacks, Bithermal Caloric stimulation would induce dizziness similar to that experienced by VS patients with chronic symptoms of imbalance, producing a priming effect for the patient’s anxiety, and this would correlate with their reported balance symptoms and dizziness handicap, consistent with the vicious cycle that Yardley et al. (8) described.

Consistent with our hypothesis, post-resection patients with a fixed, vestibular deficit developed an increase in state anxiety when presented with a vestibular stimulus. In a subsequent group of patients with varying vestibular deficits (VS *in situ*), there was a significant correlation between state anxiety during vestibular stimulation and chronic balance symptoms and handicap. Patients with balance symptoms and handicap presented with increased state anxiety after vestibular stimulation when compared with those that had no handicap or balance symptoms. To our knowledge, this is the first study in VS patients producing evidence that a priming effect linked to state anxiety may exist reinforcing chronic symptoms of imbalance and resulting handicap.

These findings add to the growing evidence that anxiety and chronic balance dysfunction are intricately related. Balance problems are commonly associated with symptoms of anxiety that have been shown to be a longitudinal predictor of increasing balance symptom severity and handicap in groups of mixed neuro-otological patients (8, 9, 25). Authors have suggested that anxiety is a better predictor of patient-reported balance symptom severity in the long term as opposed to the objective vestibular deficit (26–28). Although at a mechanistic level, there are numerous factors that can influence long-term balance outcomes, anxiety triggered by pathological or physiological vestibular sensory input with activation of the stress pathways may also have a significant effect. There are, however, several factors that need to be considered in this complex relationship. At some level, during the early stages of vestibular compensation following the acute vestibular event, stress may be beneficial to the learning process involved in early recovery and rehabilitation (16). It is, however, postulated that anxiety-related stress could have an altogether different, detrimental effect, which becomes increasingly pronounced within the first week – 6-month period after the acute vestibular event (11). Best et al. proposed that the individual pattern of psychological reaction after the onset of the vestibular disorder leads to symptom persistency and chronicity (23, 29). Yardley describes a reciprocal predictive relationship between handicap, emotional distress and somatic symptoms of anxiety, suggesting that anxiety and depression resulting from actual and anticipated disability increase fears and feelings of incompetence resulting in a greater restriction of activity further identifying such patients as having a “relinquishing responsibility” style of coping as well as a fear of losing control (8, 9, 25). Heinrichs et al. found that the interaction between the fear of bodily sensations and initial severity of dizziness during an organic episode contributes to persistent psychogenic dizziness (30). Godemann et al. suggested that dysfunctional cognitions and insecure and dependent personality types explained anxiety in VN patients (31), and in a further study suggested that anxieties and fear of vertigo 6 months after an episode of VN explained 60% of the variance in the development of panic and somatoform disorder over the next 2 years (11).

Holmberg et al. have shown that in patients with phobic postural vertigo 8–12 sessions of CBT resulted in improvement in the VHQ and hospital anxiety and depression scores; however, at 1-year follow-up, the initially described improvement remained with no additional benefits (32, 33). Similar improvements in disability were reported by Edelaman (34) following three courses of CBT in newly diagnosed patients with chronic subjective dizziness. Mahoney et al. also reported improvements in disability in patients with chronic subjective dizziness following a short course of CBT at 1 month, and these improvements were sustained at 6 months with no further significant improvement (35).

Research has also shown the benefits of Selective Serotonin Reuptake Inhibitors in treating patients with chronic dizziness and anxiety (36).

Optimally identifying VS patients with chronic subjective dizziness early on and considering the above treatment options in combination with vestibular rehabilitation seem likely to offer these patients the best treatment success before the anxiety, balance symptoms, and handicap become intertwined.

## Author Contributions

Lucie Mclellan assisted with data collection. Dr. L. Mckenna assisted with write-up. Prof. MD assisted with data collection and write-up. Mr. RO assisted with data collection and write-up. Prof. MG assisted with data collection and write-up. Prof. GL assisted with write-up. Dr. D-EB assisted with data collection and write-up.

## Conflict of Interest Statement

The authors declare that the research was conducted in the absence of any commercial or financial relationships that could be construed as a potential conflict of interest.
